# The potential roles of long non‐coding RNAs in lipopolysaccharide‐induced human peripheral blood mononuclear cells as determined by microarray analysis

**DOI:** 10.1002/2211-5463.12556

**Published:** 2018-12-25

**Authors:** Anqiang Zhang, Hongxiang Lu, Dalin Wen, Jianhui Sun, Juan Du, Xu Wang, Wei Gu, Jianxin Jiang

**Affiliations:** ^1^ State Key Laboratory of Trauma, Burns and Combined Injury Institute of Surgery Research Daping Hospital Army Medical University Chongqing China; ^2^ The Second Affiliated Hospital Zhejiang University Hangzhou China

**Keywords:** inflammation, lipopolysaccharide, lncRNA, microarray, peripheral blood mononuclear cell

## Abstract

Sepsis arises from an aberrant and excessive host response to infection. Long noncoding RNAs (lncRNAs) are involved in multiple cellular functions, including inflammation and immunity. However, to date there has been no systematic attempt to identify lncRNAs whose expression is changed after the induction of the innate immune response. In this study, we profiled global lncRNA and mRNA expression changes in peripheral blood mononuclear cells (PBMCs) treated with lipopolysaccharide (LPS) using a microarray platform. Of the 40 914 lncRNAs screened, 596 were significantly upregulated and 250 were significantly downregulated (corrected *P* < 0.05) in response to LPS. Of the 34 236 mRNAs screened, 802 were upregulated and 549 were downregulated. Functional annotation analysis indicated that lncRNA‐associated differentially expressed mRNAs were primarily enriched in host immune and inflammatory responses. This study provides the first lncRNA and mRNA transcriptomic landscape of LPS‐mediated changes in human PBMCs. These findings may provide potential insights into lncRNAs involved in the immunopathology of sepsis.

**Abbreviations**
cDNA: complementary DNA
DE: differentially expressed
GO: gene ontology
IL: interleukin
KEGG: Kyoto Encyclopedia of Genes and Genomes
lncRNA: long noncoding RNA
LPS: lipopolysaccharide
NF‐κB: nuclear factor κB
NLR: NOD‐like receptor
PACER: p50‐associated COX‐2 extragenic RNA
PBMC: peripheral blood mononuclear cell
PCC: Pearson correlation coefficient
qRT‐PCR: quantitative real‐time polymerase chain reaction
RLR: RIG‐I‐like receptor
TLR: toll‐like receptor
TNF: tumor necrosis factor
T‐UCR: transcribed ultraconserved noncoding RNA

Sepsis caused by infection remains a life‐threatening multiorgan disease with high morbidity and mortality in critically ill patients. There are an estimated 31.5 million sepsis patients and 5.3 million sepsis‐related deaths annually around the world 1. There is no available method to diagnose sepsis, and no specific treatment for patients with sepsis, and management therefore relies on effective antibiotic treatment, organ function support, and infection source removal. Superiority evidence indicated that early appropriate treatment was significantly associated with improved sepsis outcomes; the ability to identify the condition early is therefore important so that effective treatment can be started early to prevent deterioration 2. However, the fast and precise detection of sepsis remains a challenge for clinicians.

The innate immune response provides the initial defense against infection threats by external pathogens through the induction of a pathological immune‐inflammation. The presence of pathogens is commonly detected by myeloid cells, including dendritic cells, macrophages, and mononuclear cells 3. Lipopolysaccharide (LPS) causes strong immune‐inflammatory responses after recognition by toll‐like receptor (TLR) 4 on immune cells, which are activated, resulting in the release of pro‐inflammatory cytokines, such as interleukin (IL) 1β, tumor necrosis factor (TNF) α, and IL‐6. Excess immune cell activation leads to a more severe immunopathology, such as sepsis and subsequent multiple organ dysfunction syndrome. Deciphering the molecular mechanisms of host responses linked to sepsis pathogenesis and prognosis is of great importance for exploring diagnostic and therapeutic tools.

Long noncoding RNAs (lncRNAs) are tentatively defined as a large class of noncoding RNA transcripts that are > 200 bases in length without open reading frames. LncRNAs were previously thought to be functionless, but over the past decade, important roles of lncRNAs have been discovered. LncRNAs can regulate gene and genome activity by regulating histone modification and chromatin remodeling, modulating DNA methylation, interacting with transcription factors and looping enhancers, and modifying expression at the post‐transcriptional level 4. Although lncRNAs are not as conserved as protein‐coding genes and microRNAs, recent studies have shown that they are involved in multiple cellular functions, including inflammation, immunity, proliferation, differentiation, migration, invasion, survival, and angiogenesis, and could also provide alternative therapeutic targets. Thus, aberrant lncRNAs have been found in a variety of human diseases, for example cancer, autoimmune disease and Alzheimer's disease 5. Recent advances have also revealed a large number of lncRNAs that are differentially expressed (DE) following the activation of innate immunity and that regulate the subsequent immune‐inflammatory response 6. In human, these lncRNAs include lnc‐DC 7, p50‐associated COX‐2 extragenic RNA (PACER) 8, MALAT1 9, TNFα‐ and hnRNPL‐related immunoregulatory long intergenic noncoding RNA (THRIL) 10, IL1β‐RBT46 11, and lnc‐IL7R 12, while studies in mice have identified lncRNA‐HOTAIR 13, lincRNA‐EPS 14, lincRNA‐Tnfaip3 15, lincRNA‐COX2 16, and AS‐IL1α 17. However, despite this evidence indicating that lncRNAs act as key regulators, there has been no systematic attempt to identify DElncRNAs after the induction of the innate immunity. The alterations in lncRNA expression that are induced by LPS and the roles of these transcripts in modulating the peripheral blood mononuclear cell (PBMC) response to infection remain unclear.

In this study, we aimed to profile global lncRNA and mRNA expression changes in PBMCs treated with LPS using a microarray platform, which may provide novel perspectives into the regulation of the inflammatory response in PBMCs by LPS‐associated lncRNAs. Furthermore, the approach also may identify some novel sepsis‐related lncRNAs and associated pathways in PBMCs that could serve as potential therapeutic targets.

## Methods

### Preparation of blood samples and LPS stimulation

Human peripheral blood was collected from 12 healthy volunteers. PBMCs were separated using Hypaque‐Ficoll density gradient centrifugation (TBD, Tianjin, China) according to the manufacturer's protocol. PBMCs were resuspended in RPMI 1640 containing 10% (v/v) fetal bovine serum and incubated with the absence or presence of 100 ng·mL^−1^ *Escherichia coli* LPS (O55:B5; Sigma‐Aldrich, St Louis, MO, USA) for 4 h at 37 °C. This study was approved by the Ethics Committee of Army Military Medical University (registration number TMMU2012009, Chongqing, China) and carried out in accordance with the Declaration of Helsinki. Written informed consent was obtained from all participants.

### RNA extraction and quality control

Total RNA was extracted from 12 pairs of PBMCs using TRIzol^®^ reagent (Thermo Fisher Scientific, Inc., Waltham, MA, USA) and purified with the NucleoSpin^®^ RNA clean‐up Kit (Macherey‐Nagel, Düren, Germany) according to the manufacturer's protocol. Purity and concentration were detected using a NanoDropND‐2000 spectrophotometer (Thermo Fisher Scientific). RNA integrity was assessed by 1% formaldehyde denaturing gel electrophoresis.

### Microarray detection

Five hundred nanograms of RNA was first reverse transcribed into complementary DNA (cDNA). The cDNA was amplified with random primers and labeled with Cy3‐dCTP (green) and Cy5‐dCTP (red) using a CapitalBio cRNA Amplification and Labeling Kit (CapitalBio, Beijing, China) according to the manufacturer's protocol. The labeled cDNA was hybridized on the Agilent human lncRNA microarray v4.0 (Agilent Technologies, Santa Clara, CA, USA), which contained 40 914 lncRNA and 34 236 coding transcript probes. The lncRNA probes were designed according to discovered lncRNAs from GENCODE, ENSEMBL, Human LincRNA Catalog, RefSeq, UCSC, NRED, LNCipedia, H‐InvDB, RNAdb, UCR, and the Chen Ruisheng laboratory.

### Differential expression analysis of lncRNAs and mRNAs

The microarray data were analyzed for normalization, summarization, and quality control by using genespring software v13.0 (Agilent). The expression signal value was log2 transformed and median centered by gene using the adjust data function of cluster 3.0 software (University of Tokyo, Human Genome Center, Tokyo, Japan). Significantly differentially expressed lncRNAs and mRNAs were defined as having a Benjamini–Hochberg corrected *P* value < 0.05 and an absolute fold change ≥ 2.0. Two‐dimensional hierarchical clustering was used to arrange the samples into groups based on their expression levels. The findings were illustrated with a volcano plot and scatter plots of the altered lncRNAs and mRNAs. DElncRNA classification was carried out to explore the potential function. All array experiments were conducted at CapitalBio Corp., Beijing, China.

### Correlation and coexpression analysis

The correlations between the expression levels of DElncRNAs and DEmRNAs were calculated by Pearson correlation coefficients (PCCs), which range from −1.00 to 1.00, and larger absolute PCC values indicate stronger interactions. In the present study, the pairs with PCCs > 0.90 or < −0.90 and *P* values < 0.05 were chosen to build the coexpression network. In this network, the number of links from each lncRNA to a mRNA or another lncRNA was calculated and defined as the degree of centrality. A higher degree for a lncRNA indicated that the lncRNA played a more important role in the network.

### GO and KEGG pathway analyses

Gene ontology (GO) analysis is a functional analysis of DEmRNAs that is performed by connecting the mRNAs with the GO categories derived from Gene Ontology (www.geneontology.org). Gene functions were classified into cellular component, biological process, and molecular function. Next, the latest Kyoto Encyclopedia of Genes and Genomes (KEGG) database (www.genome.jp/kegg) was used to determine the involvement of DEmRNAs in different biological pathways. The recommended *P* value (hypergeometric *P* value) cutoff is 0.05.

### Quantitative real‐time PCR validation

The expression levels of DElncRNAs were confirmed by quantitative real‐time PCR (qRT‐PCR). In brief, total RNA extracted from PBMCs was reverse transcribed into cDNA using a PrimeScript™ RT Reagent kit (Takara Biotechnology Co., Ltd., Dalian, China) according to the manufacturer's protocol. Following the cDNA synthesis, the quantitative PCR amplifications were performed using the SYBR Green Real time PCR Master Mix (Toyobo Co., Ltd, Osaka, Japan). The relative expression levels of the lncRNAs were quantified using the $2^{-\Delta \Delta C_{T}}$ method, and glyceraldehyde 3‐phosphate dehydrogenase was used for normalization. The data represent the means of three experiments.

### Statistical analysis

Data are presented as means ± standard deviation (SD) of at least three independent experiments. Statistical significance was assessed using a two‐tailed Student's *t* test for quantitative data. *P* < 0.05 was taken to indicate statistical significance. All analyses were performed using spss 11.5 (SPSS, Inc., Chicago, IL, USA).

## Results

### Quality assessment of lncRNA and mRNA data

All 24 samples included had a 2 : 1 28S : 18S rRNA intensity ratio and *A*_260_/*A*_280_ ratios > 1.7, verifying their RNA purity and integrity (Fig. S1A). The distribution of fluorescence intensities was visualized using box–whisker plots and revealed similar normalized log2 ratios for lncRNAs and mRNAs within each sample; accordingly, the quality of the array data was comparable across samples (Fig. S1B).

To investigate the possible regulation functions of lncRNAs in LPS‐induced PBMCs, the expression level of lncRNAs and mRNAs was estimated using microarray analysis. In total, the signals of 21 518 lncRNAs from authoritative data sources were captured in the lncRNA microarray, with most of their lengths between 200 and 3000 nucleotides. The highly expressed lncRNAs with signal intensities above 90 000 are presented in Fig. S2A. Among those lncRNAs, TCONS_00006919, TCONS_00006918, and ENST00000451766.1 were the most abundant in human PBMCs. There was no significant difference in highly expressed lncRNA species after LPS treatment.

**Figure 1:**
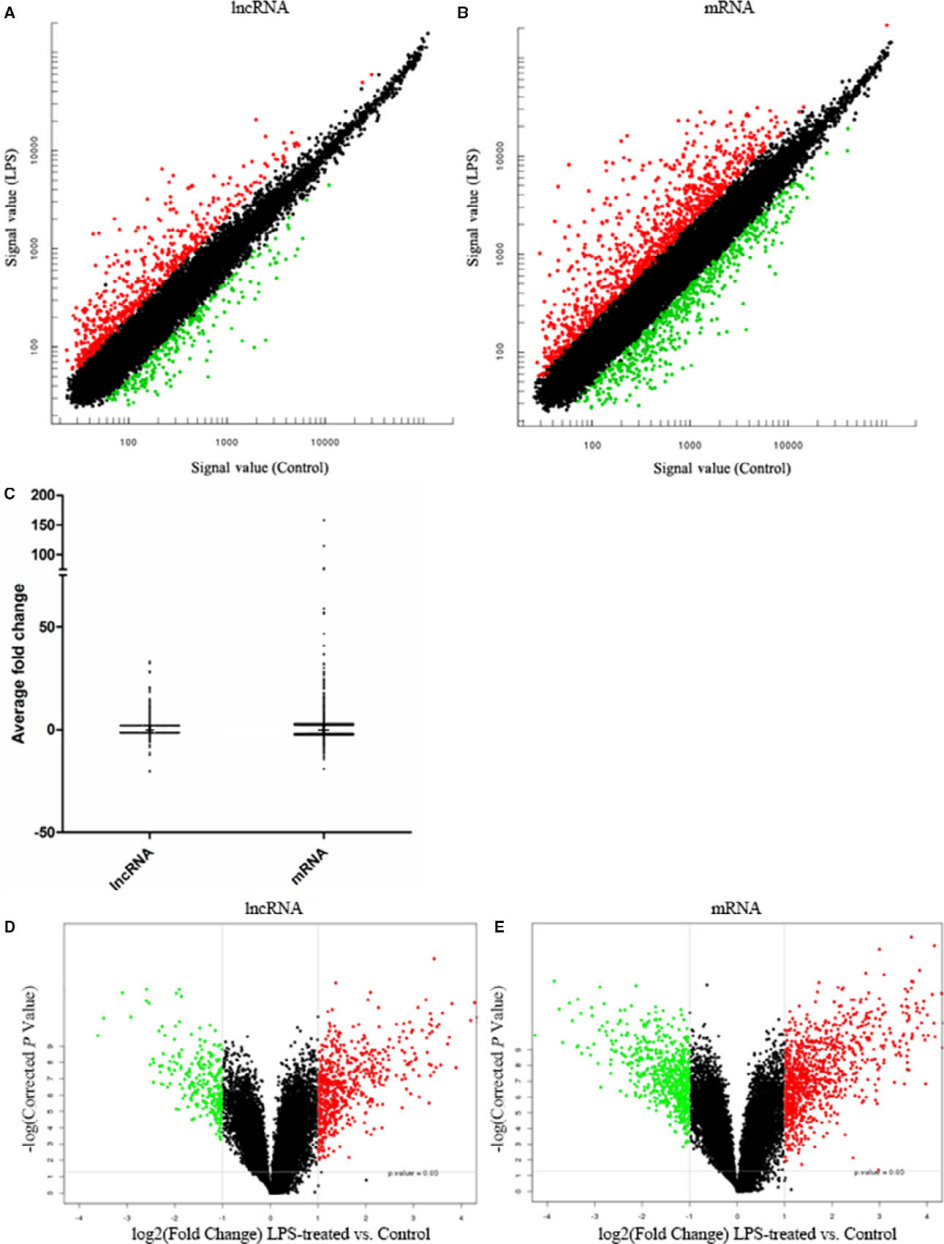
LncRNA and mRNA expression profiles in PBMCs treated with LPS (100 ng·mL^−1^) *vs* controls. (A, B) Scatter plots comparing the variations in lncRNA and mRNA expression. The values plotted are the averaged normalized signal values (log2 scaled) for the control (*x*‐axis) and the LPS treatment (*y*‐axis) groups. The red and green points indicate a greater than 2‐fold change between the two groups. (C) Box–whisker plots (10th and 90th percentiles) showing the average fold changes of lncRNAs and mRNAs. The mean intensity is denoted by ‘+’. (D, E) Volcano plots detailing the magnitude of expression differences. The vertical gray lines correspond to 2.0‐fold upregulation and 2.0‐fold downregulation of expression. The horizontal gray line indicates a *P* value of ≤ 0.05. The red and green points represent lncRNAs and mRNAs with statistically significant differential expression.

Scatter plots were used to show variation in lncRNAs (Fig. 1A) and mRNAs (Fig. 1B) expression between LPS‐treated and vehicle‐treated PBMCs. In general, the average fold changes were similar between DElncRNAs and DEmRNAs under the same conditions (Fig. 1C). A volcano plot revealed 596 and 250 lncRNAs that were significantly upregulated and downregulated, respectively, in LPS‐treated PBMCs compared to the expression levels in control samples (Fig. 1D; fold change ≥ 2.0 and corrected *P* < 0.05). The length of DElncRNAs ranged from 60 to 32.8 kb. Specifically, linc‐HELT‐3 (Chromosome 4, RNA length: 826 bp) was the most markedly upregulated lncRNA (~ 33‐fold), and AC011899.9 (Chromosome 7, RNA length: 4302 bp) was the most markedly downregulated lncRNA (~ 20‐fold) in LPS‐treated PBMCs. The top 10 up‐ and downregulated DElncRNAs according to the fold change in expression are listed in Table 1. Transcript fluxes after LPS stimulation were also noted. Among the detected 17 703 mRNAs, 802 were significantly upregulated, and 549 were downregulated (Fig. 1E; fold change ≥ 2.0 and corrected *P* < 0.05). Immunoresponsive 1 homolog (mouse) (IRG1), the important component of the signal pathway network in sepsis, was the most markedly dysregulated mRNA (~ 158‐fold up). Two‐dimensional hierarchical clustering analysis of the 846 DElncRNAs (Fig. S2B) and 1351 DEmRNAs (Fig. S2C) was then performed. The clustering provided clear separation into LPS‐treated group and sham group clusters, indicating that these lncRNAs and mRNAs were differentially expressed after LPS stimulation.

**Table 1:** Ten most differentially expressed (up‐ and downregulated) lncRNAs in LPS‐treated (100 ng·mL^−1^) *vs* PBS‐treated PBMCs. *P* (corr) was calculated by Benjamini–Hochberg false discovery rate

lncRNA name	lncRNA ID	Length	Chr	FC	*P* (corr)
Upregulated lncRNAs (top 10)					
linc‐HELT‐3	TCONS_00008360	826	4	33.07	1.57E‐09
TSG‐37 mRNA fragment	ASO2257	363	2	32.16	9.42E‐11
RP11‐519G16.5	ENST00000559553.1	729	15	28.43	2.12E‐12
RP11‐44K6.2	ENST00000520185.1	102	8	27.65	5.58E‐10
RP11‐190C22.9	ENST00000609385.1	439	3	20.54	7.5E‐09
RP13‐452N2.1	ENST00000462083.2	2139	7	20.21	5.13E‐09
KB‐1507C5.4	ENST00000517983.1	563	8	19.42	1.24E‐09
linc‐DGCR6‐1	TCONS_00029753	893	22	18.37	6.72E‐09
linc‐IRX3‐4	TCONS_00024668	308	16	14.85	7.51E‐07
LOC100505869	XR_110454.2	529	10	13.15	7.00E‐08
Downregulated lncRNAs (top 10)					
AC011899.9	ENST00000438047.1	4302	7	20.47	1.94E‐10
XR_171041.1	XR_171041.1	557	7	20.24	3.63E‐10
NR_026754	uc010qoz.1	686	10	12.22	3.07E‐08
CTD‐2540B15.11	ENST00000589932.1	659	19	11.23	5.37E‐09
AL163636.6	ENST00000553909.1	1498	14	8.57	5.08E‐10
RP11‐702H23.6	ENST00000530510.1	609	11	7.57	4.83E‐09
RP11‐1055B8.4‐002	ENST00000608155.1	414	17	6.04	1.01E‐09
RP11‐1055B8.4‐001	ENST00000571724.2	1561	17	6.03	3.59E‐10
RP11‐356N1.2	ENST00000419428.1	432	1	5.82	1.29E‐09
linc‐ACTL8‐1	TCONS_00000852	846	1	5.50	3.12E‐07

### LncRNA classification and subgroup analysis

**Figure 2:**
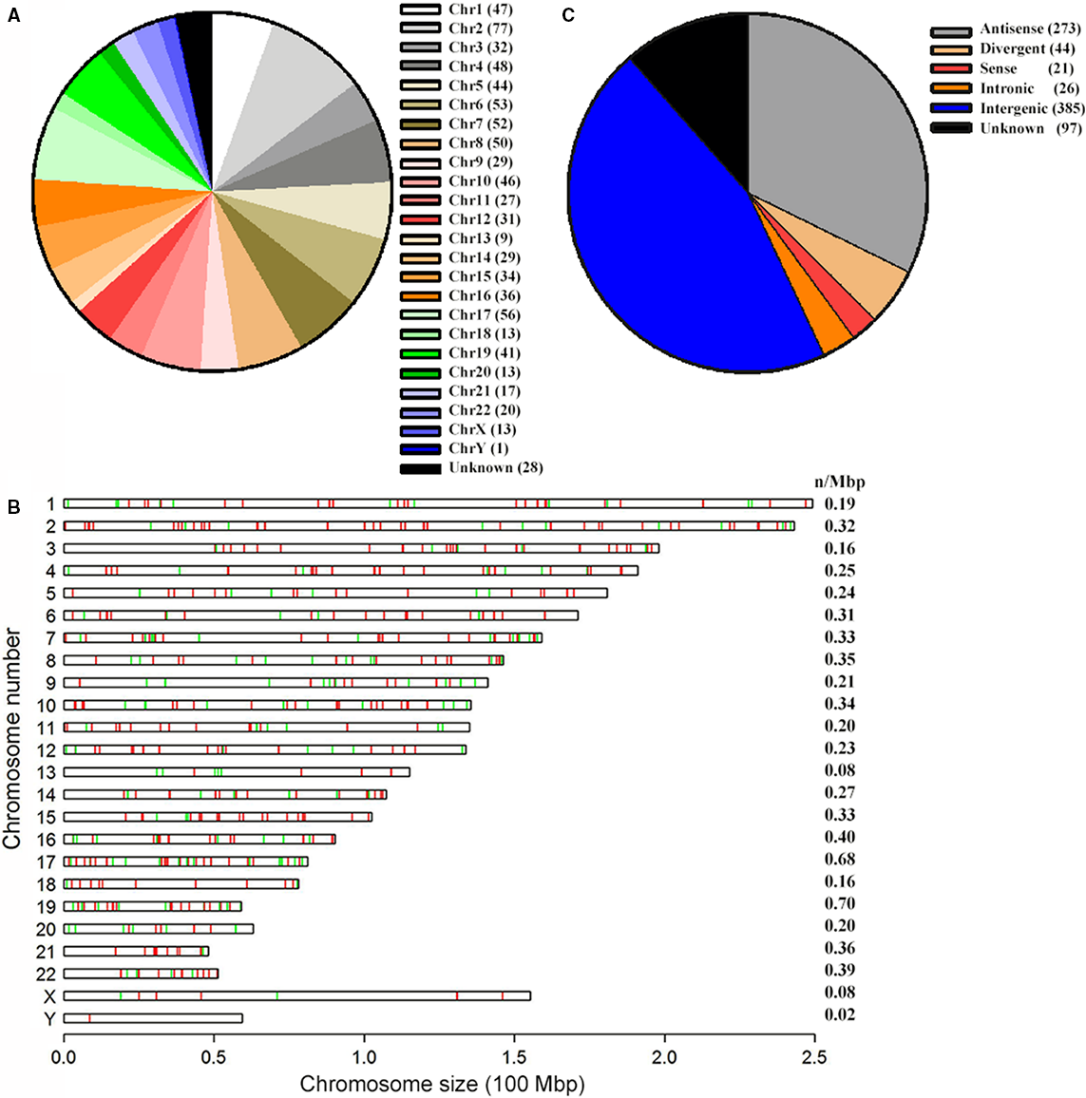
Distribution, location, and classification of DElncRNAs in PBMCs treated with LPS (100 ng·mL^−1^) *vs* controls. (A, B) Illustration of the numbers (A) and chromosomal locations (B) of DElncRNAs on different chromosomes. (C) Bar graph representing the types of DElncRNAs depending upon their genomic locations.

The DElncRNAs were further classified according to their features, including genome location, contact, and exerted effect on DNA. As illustrated, although DElncRNAs of PBMCs modulated by LPS were abundant and located into each of chromosomes, chromosomes 2, 6, and 17 had the most number of DElncRNAs (Fig. 2A). Further exploration revealed that these DElncRNAs were markedly clustered when they were located along the entire length of the chromosomes (Fig. 2B); chromosomes 17 and 19 had the most DElncRNAs per Mbp (*n* = 0.68 Mbp^−1^). Subgroup analysis of DElncRNAs showed that the majority (~ 46%) of DElncRNAs were intergenic, followed by antisense and divergent lncRNAs (Fig. 2C), which helps identify the functional relationships between lncRNAs and their associated protein‐coding genes. We also identified intronic and sense‐overlapping lncRNAs (Fig. 2C). Transcribed ultraconserved noncoding RNAs (T‐UCRs) are an important group of sequences that are 100% conserved among orthologous regions of the human, rat, and mouse genomes. Of 407 detected T‐UCRs, nine T‐UCRs were differentially expressed in LPS‐stimulated PBMCs, including six upregulated and three downregulated T‐UCRs.

### Pathway and GO analysis of dysregulated mRNAs

KEGG pathway analysis indicated several relevant signal pathways. In brief, the LPS‐induced DElncRNAs are involved in the nuclear factor κB (NF‐κB) signaling pathway, the toll‐like receptor (TLR) signaling pathway, the NOD‐like receptor (NLR) signaling pathway, the TNF signaling pathway, cytokine–cytokine receptor interaction, and the RIG‐I‐like receptor (RLR) signaling pathway (Table 2).

**Table 2:** KEGG pathway enrichment of dysregulated mRNAs in PBMCs in response to LPS (100 ng·mL^−1^). *P* (corr) was calculated by Benjamini–Hochberg false discovery rate

Item	Details	Gene count	*P* (corr)
hsa04064	NF‐κB signaling pathway	33	9.42E‐07
hsa05164	Influenza A	47	1.65E‐06
hsa04060	Cytokine–cytokine receptor interaction	60	4.51E‐06
hsa05134	Legionellosis	24	8.53E‐06
hsa04380	Osteoclast differentiation	34	2.57E‐04
hsa05162	Measles	34	2.57E‐04
hsa04621	NOD‐like receptor signaling pathway	20	6.63E‐04
hsa05144	Malaria	18	6.80E‐04
hsa05168	Herpes simplex infection	40	7.79E‐04
hsa04620	Toll‐like receptor signaling pathway	27	8.59E‐04
hsa04668	TNF signaling pathway	29	8.59E‐04
hsa05133	Pertussis	22	0.0017
hsa05323	Rheumatoid arthritis	24	0.0021
hsa05152	Tuberculosis	37	0.0023
hsa05140	Leishmaniasis	21	0.0025
hsa05143	African trypanosomiasis	13	0.0055
hsa04623	Cytosolic DNA‐sensing pathway	17	0.0071
hsa05132	*Salmonella* infection	21	0.0104
hsa05150	*Staphylococcus aureus* infection	16	0.0115
hsa04622	RIG‐I‐like receptor signaling pathway	18	0.0119

Through the GO analysis (Table 3), the DEmRNAs were cataloged into the three categories, containing biological processes, cellular components, and molecular functions. The GO terms that were most significantly associated with the dysregulated mRNAs included defense response, side of membrane, and cytokine activity.

**Table 3:** GO analysis of DEmRNAs. *P* (corr) was calculated by Benjamini Hochberg false discovery rate

GO ID	GO term	*P* (corr)	Gene count
Biological process			
GO:0006952	Defense response	2.74E‐23	276
GO:0006955	Immune response	1.09E‐19	251
GO:0006954	Inflammatory response	7.91E‐18	138
GO:0051707	Response to other organisms	7.22E‐17	149
GO:0043207	Response to external biotic stimulus	7.22E‐17	149
Cellular component			
GO:0098552	Side of membrane	1.67E‐4	55
GO:0009897	External side of plasma membrane	1.85E‐4	44
GO:0045121	Membrane raft	0.0018	42
GO:0009986	Cell surface	0.014	86
GO:0005887	Integral component of plasma membrane	0.031	149
Molecular function			
GO:0005125	Cytokine activity	1.86E‐05	46
GO:0005126	Cytokine receptor binding	8.93E‐05	49
GO:0042379	Chemokine receptor binding	6.93E‐04	18
GO:0008009	Chemokine activity	0.0023	15
GO:0004896	Cytokine receptor activity	0.0053	21

### DElncRNA/DEmRNA coexpression network

**Figure 3:**
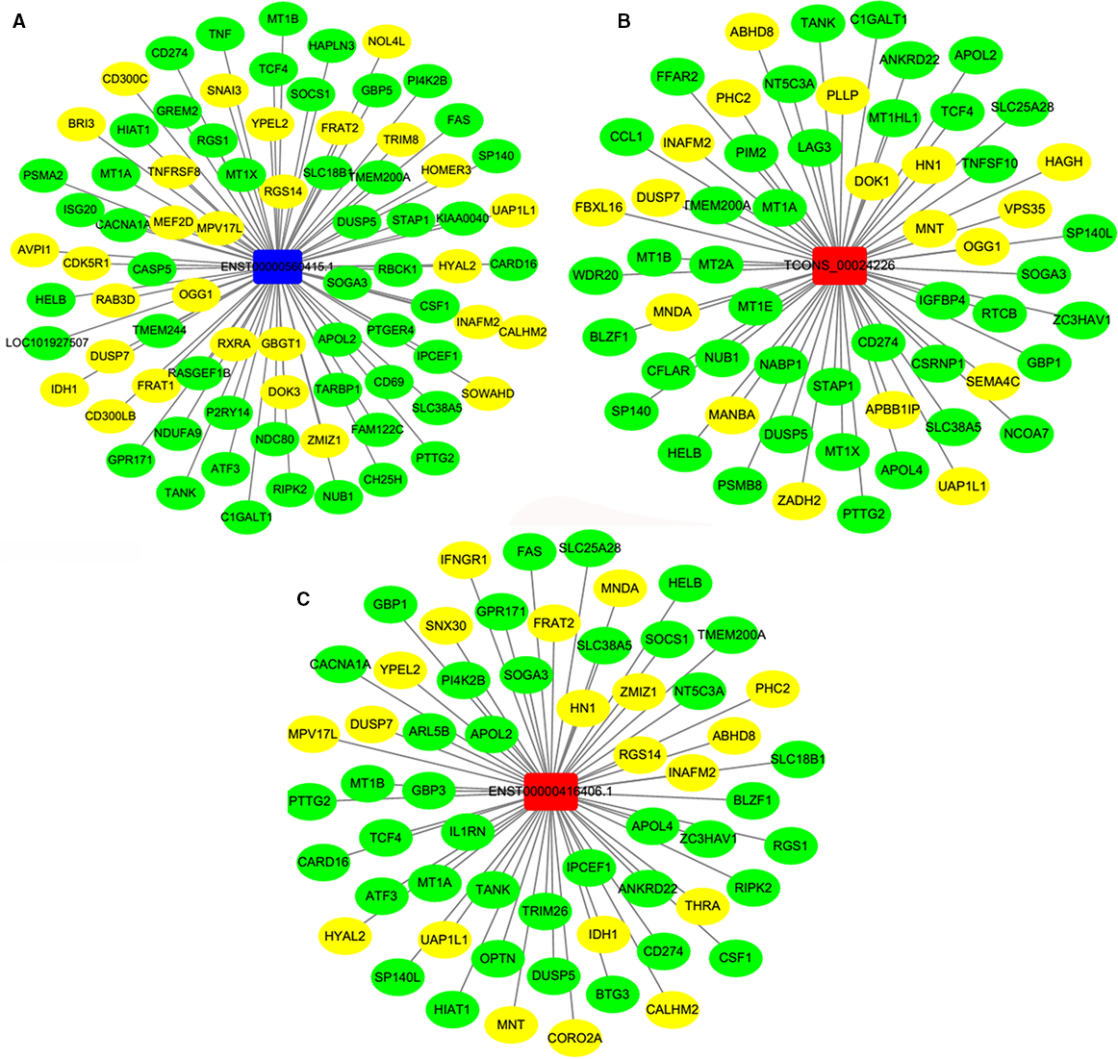
Subnetworks of DElncRNA–DEmRNA coexpression. (A) The subnetwork of lnc‐INAFM2‐1; (B) the subnetwork of linc‐MT1B‐1; and (C) the subnetwork of lnc‐GTPBP1‐2. Circular nodes represent DEmRNAs, and rectangular nodes represent DElncRNAs. Green indicates upregulated DEmRNAs, and yellow indicates downregulated DEmRNAs. Red indicates upregulated DElncRNAs, and blue indicates downregulated DElncRNAs.

To investigate the potential interactions of DElncRNAs and DEmRNAs in the inflammatory response, PCCs indicating the coexpression relationships between 846 DElncRNAs and 1351 DEmRNAs were calculated based on the expression levels of the lncRNAs and mRNAs. DElncRNA–DEmRNA coexpression pairs with PCC > 0.9 or < −0.9 were selected for further analysis. In Fig. S3A, a total of 434 lncRNAs and 547 mRNAs were involved in the coexpression network, which consisted of 981 nodes and 2976 connections; 85 lncRNAs and 243 mRNAs were involved in the central biggest cluster through network clustering (Fig. S3B), and were associated with the cytokine–cytokine receptor interaction, mitogen‐activated protein kinase signaling pathway, and the Janus kinase (JAK)–signal transducers and activators of transcription (STAT) signaling pathway. lnc‐INAFM2‐1 (ENST00000560415.1) (Fig. 3A), linc‐MT1B‐1 (TCONS_00024226) (Fig. 3B), and lnc‐GTPBP1‐2 (ENST00000416406.1) (Fig. 3C) were the hub DElncRNAs in the network, which had high connectivity with DEmRNAs.

### Quantitative real‐time PCR validation

To verify the data obtained from microarray profiling, we performed qRT‐PCR on a subset of 10 randomly chosen lncRNAs, which represented seven upregulated lncRNAs and three downregulated lncRNAs (Table 4). The results of qRT‐PCR indicated either up‐ or downregulated transcription, which correlated with up‐ or downregulation in the microarray. This validation demonstrated that the results obtained from microarray and qRT‐PCR analysis are highly concordant. qRT‐PCR tests confirmed the quality and robustness of the results.

**Table 4:** LncRNAs that were differentially expressed in LPS‐induced PBMCs, as determined by microarray, and selected for validation studies. *P* was calculated by two‐tailed Student's *t* test

Seq name	Gene symbol	Class	Array		qRT‐PCR	
			Fold change	*P*	Fold change	*P*
TCONS_00008360	linc‐HELT‐3	Intergenic	33.07 ± 11.97	3.24E‐12	120.92 ± 81.36	3.44E‐04
ENST00000559553.1	RP11‐519G16.5	Antisense	28.43 ± 3.83	1.37E‐16	43.13 ± 13.33	4.47E‐05
ENST00000520185.1	RP11‐44K6.2	Sense	27.65 ± 8.05	7.31E‐13	41.11 ± 20.87	1.88E‐04
ENST00000609385.1	RP11‐190C22.9	Antisense	20.54 ± 7.54	3.35E‐11	23.20 ± 14.31	4.50E‐04
XR_110454.2	XR_110454.2	Divergent	13.15 ± 6.18	7.50E‐10	18.63 ± 9.89	0.002
TCONS_00026848	linc‐FAM187B‐2	Intergenic	10.76 ± 6.37	3.92E‐08	3.52 ± 1.15	1.18E‐04
TCONS_00024667	linc‐IRX3‐4	Intergenic	10.07 ± 4.37	1.35E‐08	4.30 ± 1.91	0.001
XR_171041.1	XR_171041.1	Antisense	−20.24 ± 5.42	3.59E‐13	−16.59 ± 4.58	1.01E‐04
uc010qoz.1	uc010qoz.1	Antisense	−12.22 ± 5.86	2.31E‐10	−17.13 ± 5.31	1.95E‐04
ENST00000589932.1	CTD‐2540B15.11	Antisense	−11.24 ± 3.76	1.99E‐11	−18.70 ± 4.75	1.45E‐05

## Discussion

The molecular mechanisms of associated lncRNA responsible for the LPS‐induced activation of the immune inflammatory response in PBMCs remain largely undefined. An accumulating body of evidence has indicated that lncRNAs are involved in inflammatory processes 18. In this study, we investigated lncRNA and mRNA expression profiles in response to LPS‐induced PBMCs and identified potential genetic targets of lncRNAs. Our findings provide novel insights into the key role of lncRNAs during the activation of the innate immune response. KEGG pathway analysis indicated that LPS affects several important lncRNA‐associated pathways, such as cytokine–cytokine receptor interactions and the NF‐κB, NLR, TLR, and TNF signaling pathways. These findings could potentially be utilized for diagnostic and therapeutic purposes in patients with LPS‐associated illnesses.

In the present study, of the 40 914 lncRNAs screened, we identified 596 and 250 that were significantly up‐ and downregulated, respectively, in PBMCs treated with LPS compared to the expression levels in vehicle‐treated control samples. In the same PBMC samples, 802 mRNAs were upregulated, and 549 mRNAs were downregulated among 34 236 protein‐coding transcripts. According to their position and direction of transcription in relation to neighboring protein‐coding genes, lncRNAs can be further categorized into five broad subcategories: antisense, sense, intergenic, intronic, and bidirectional 19. Approximately half of DElncRNAs in LPS‐treated PBMCs belonged to the intergenic subcategory, which describes lncRNAs that are located within the genomic interval between two protein‐coding genes and could regulate functional gene expression through interaction with various chromatin‐modifying complexes or microRNAs 20, 21. These classifications may help predict their function in the LPS‐induced inflammation response. LncRNAs function via a variety of mechanisms; however, a common and important function of lncRNAs is to alter nearby protein‐coding gene expression by affecting the transcription process or directly playing an enhancer‐like role 22. For example, IIott *et al*. 11 demonstrated that enhancer‐like lncRNAs are associated with the innate immune response in human primary monocytes. Furthermore, their results demonstrated that 40 canonical lncRNAs, 76 enhancer RNAs (eRNAs), 35 regions of bidirectional transcription (RBTs) and 65 antisense lncRNAs are differentially expressed in response to LPS. Crucially, the knockdown of nuclear‐localized transcripts, IL1β‐eRNA and IL1β‐RBT46(+), surrounding the IL‐1β locus attenuates the release of the inflammatory cytokines CXCL8 and IL‐1β.

GO category and KEGG pathway annotation was used to investigate the potential regulatory roles of DElncRNAs, and demonstrated that the transcripts downregulated and upregulated by lncRNAs were associated with cellular components, biological processes, and molecular functions; these functions were associated with 23 signal pathways, for example, ‘NF‐κB signaling pathway’, ‘NLR signaling pathway’, ‘TLR signaling pathway’, ‘TNF signaling pathway’, and ‘cytokine–cytokine receptor interaction’, which indicated that the DElncRNAs were predominantly associated with the regulation of multiple inflammatory associated genes. Furthermore, these pathways are also associated with the initiation and development of sepsis. In summary, this investigation provides genome‐wide screening for the lncRNA and mRNA profile of LPS‐mediated inflammation in PBMCs. These findings may provide new targets of LPS in immune cells and sepsis‐associated immune responses.

Subsequently, 10 altered lncRNAs were randomly selected, and their expression levels of microarray were validated through qRT‐PCR. The changes in lncRNA expression levels were concordant with the microarray data. Generally, data from microarray analysis require confirmation by qRT‐PCR, which is considered to be more accurate.

## Conclusions

In this study, lncRNA and mRNA expression levels in PBMCs after LPS stimulation were analyzed using microarrays, which provided a novel and interesting foundation for improving our understanding of the association between PBMC lncRNA homeostasis and sepsis pathology. However, the findings of the present study represent a starting point for the function of lncRNAs in sepsis. Further investigations are still required to evaluate the biological functions of these identified lncRNAs and these signaling pathways with regard to their roles in the development and progression of sepsis, which are likely to reveal novel diagnosis and therapeutic targets.

## Conflict of interest

The authors declare no conflict of interest.

## Author contributions

AZ and HL were the main researchers for this study and contributed to writing the manuscript. JD, XW, and WG were involved in the collecting of blood samples. DW and JS did the technical work. JJ planned the study, wrote the protocol, was involved in the data analyses and revised the manuscript. All authors read and approved the final manuscript.

## Supporting information


**Fig. S1.** Quality assessment of RNA, lncRNA and mRNA data. (A) Image of a denaturing agarose gel (1%) used to assess RNA integrity and genomic DNA contamination. The 28S and 18S rRNA bands were clear and intact. The larger rRNA (28S) bands were more intense than the corresponding smaller rRNA (18S) bands (almost double). The table shows the absorbance ratios for wavelengths of 260 nm/280 nm and 260 nm/230 nm and the concentration and quantity of RNA used for the array. (B) Box–whisker plots (10th and 90th percentiles) showing the normalized intensity for the 24 study samples to quickly visualize the distribution of our dataset. The mean intensity is denoted by ‘–’. Even numbers represent the control group, and odd numbers represent the LPS‐treated (100 ng·mL^−1^) group.

**Fig. S2.** Heat map and hierarchical clustering of differences in lncRNA and mRNA expression in PBMCs treated with LPS (100 ng·mL^−1^) *vs* controls. (A) The highly expressed lncRNAs with signal intensities above 90 000, as detected by microarray. (B,C) Dendrogram showing the relationships among the sample expression levels. Hierarchical clustering was performed based on the term ‘differentially expressed lncRNAs and mRNAs’ and revealed distinguishable lncRNA and mRNA expression profiles among the samples.

**Fig. S3.** (A) Coexpression network of 547 DEmRNAs and 434 DElncRNAs. (B) The central largest cluster is of 243 DEmRNAs and 85 DElncRNAs. Circular nodes represent DEmRNAs, and rectangular nodes represent DElncRNAs. Green indicates upregulated DEmRNAs, and yellow indicates downregulated DEmRNAs. Red indicates upregulated DElncRNAs, and blue indicates downregulated DElncRNAs.
