# Supplementary figures and images for: The potential roles of long non‐coding RNAs in lipopolysaccharide‐induced human peripheral blood mononuclear cells as determined by microarray analysis
Source: FEBS Open Bio. 2018 Dec 25;9(1):148–58. doi: 10.1002/2211-5463.12556 (PMC6325599; doi:10.1002/2211-5463.12556)

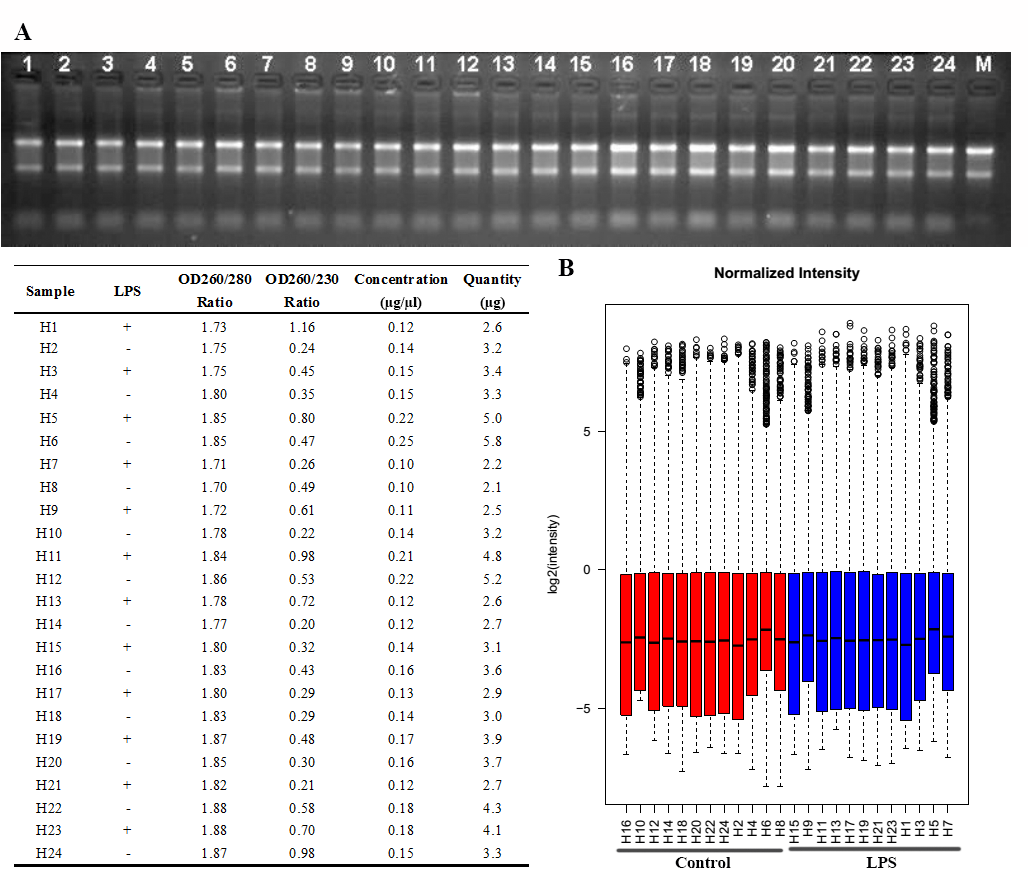

Supplement: Supplementary file 1 — Fig. S1. Quality assessment of RNA, lncRNA and mRNA data. (A) Image of a denaturing agarose gel (1%) used to assess RNA integrity and genomic DNA contamination. The 28S and 18S rRNA bands were clear and intact. The larger rRNA (28S) bands were more intense than the corresponding smaller rRNA (18S) bands (almost double). The table shows the absorbance ratios for wavelengths of 260 nm/280 nm and 260 nm/230 nm and the concentration and quantity of RNA used for the array. (B) Box–whisker plots (10th and 90th percentiles) showing the normalized intensity for the 24 study samples to quickly visualize the distribution of our dataset. The mean intensity is denoted by ‘–’. Even numbers represent the control group, and odd numbers represent the LPS‐treated (100 ng·mL−1) group. [file FEB4-9-148-s001.tif]

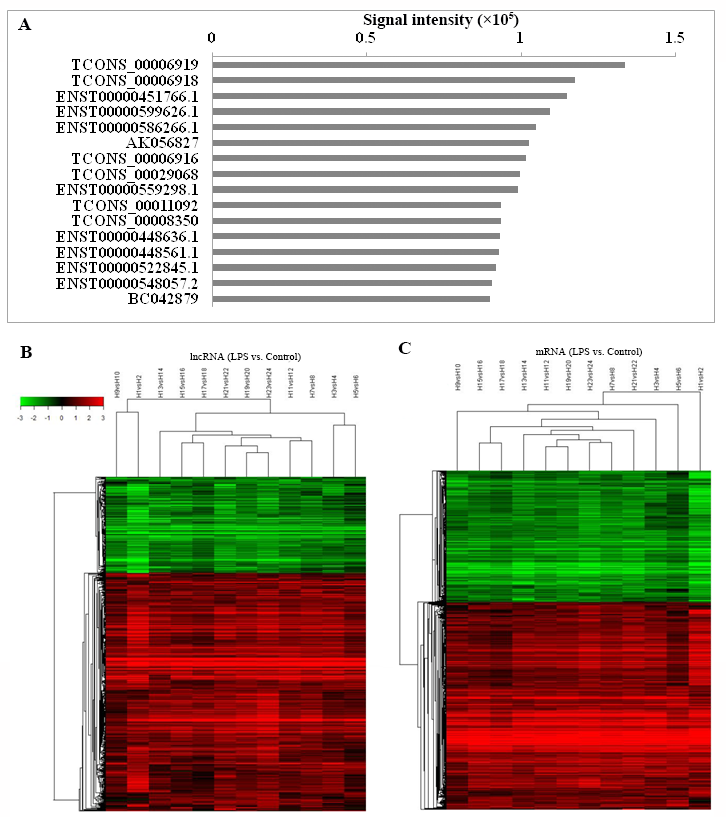

Supplement: Supplementary file 2 — Fig. S2. Heat map and hierarchical clustering of differences in lncRNA and mRNA expression in PBMCs treated with LPS (100 ng·mL−1) vs controls. (A) The highly expressed lncRNAs with signal intensities above 90 000, as detected by microarray. (B,C) Dendrogram showing the relationships among the sample expression levels. Hierarchical clustering was performed based on the term ‘differentially expressed lncRNAs and mRNAs’ and revealed distinguishable lncRNA and mRNA expression profiles among the samples. [file FEB4-9-148-s002.tif]

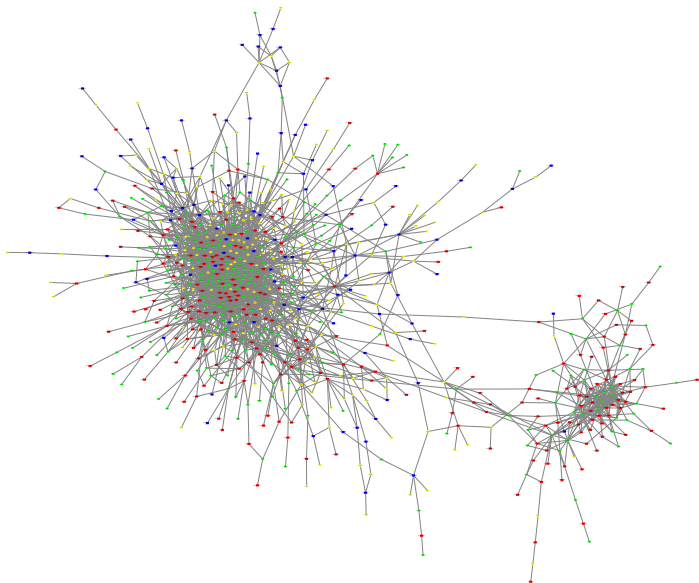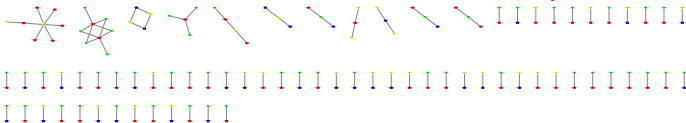

Supplement: Supplementary file 3 — Fig. S3. (A) Coexpression network of 547 DEmRNAs and 434 DElncRNAs. (B) The central largest cluster is of 243 DEmRNAs and 85 DElncRNAs. Circular nodes represent DEmRNAs, and rectangular nodes represent DElncRNAs. Green indicates upregulated DEmRNAs, and yellow indicates downregulated DEmRNAs. Red indicates upregulated DElncRNAs, and blue indicates downregulated DElncRNAs. [file FEB4-9-148-s003.pdf]

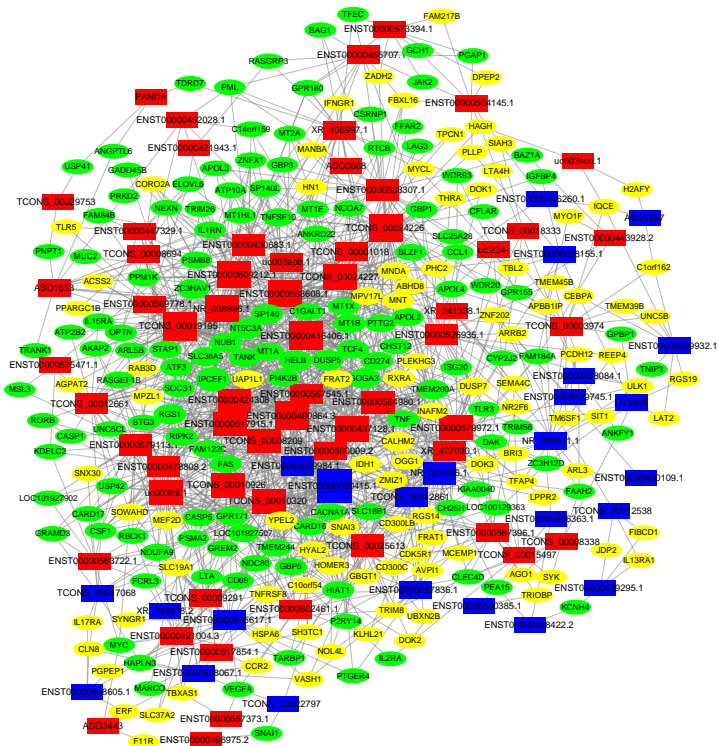

Supplement: Supplementary file 4 [file FEB4-9-148-s004.pdf]
